# Intracellular transport and regulation of transcytosis across the blood–brain barrier

**DOI:** 10.1007/s00018-018-2982-x

**Published:** 2018-12-06

**Authors:** Roberto Villaseñor, Josephine Lampe, Markus Schwaninger, Ludovic Collin

**Affiliations:** 1 Roche Pharma Research and Early Development (pRED), Pharmaceutical Sciences, Roche Innovation Center, Basel, Switzerland; 2 Institute for Experimental and Clinical Pharmacology and Toxicology, University of Lübeck, Lübeck, Germany; 3 DZHK (German Research Centre for Cardiovascular Research), Partner Site Hamburg/Lübeck/Kiel, Germany; 4 Roche Pharma Research and Early Development (pRED), Neuro-Immunology, Roche Innovation Center, Basel, Switzerland

**Keywords:** Endocytosis, Membrane trafficking, Endothelial cells, Neuroscience

## Abstract

The blood–brain barrier is a dynamic multicellular interface that regulates the transport of molecules between the blood circulation and the brain parenchyma. Proteins and peptides required for brain homeostasis cross the blood–brain barrier via transcellular transport, but the mechanisms that control this pathway are not well characterized. Here, we highlight recent studies on intracellular transport and transcytosis across the blood–brain barrier. Endothelial cells at the blood–brain barrier possess an intricate endosomal network that allows sorting to diverse cellular destinations. Internalization from the plasma membrane, endosomal sorting, and exocytosis all contribute to the regulation of transcytosis. Transmembrane receptors and blood-borne proteins utilize different pathways and mechanisms for transport across brain endothelial cells. Alterations to intracellular transport in brain endothelial cells during diseases of the central nervous system contribute to blood–brain barrier disruption and disease progression. Harnessing the intracellular sorting mechanisms at the blood–brain barrier can help improve delivery of biotherapeutics to the brain.

## Introduction to the cellular architecture of the blood–brain barrier

The blood–brain barrier (BBB) is a highly selective interface between the systemic circulation and the brain parenchyma which is required for brain homeostasis and protection of the sensible neuronal environment. Electron microscopy studies in the 1960s localized the BBB to the brain vasculature and concluded that a barrier between blood and brain parenchyma is maintained by intercellular tight junctions and reduced vesicular trafficking, which limit paracellular and transcellular transport, respectively [1]. A crucial requirement for the functionality of the BBB is the intensive cross-talk between brain endothelial cells (BECs), pericytes and astrocytes, which together with the extracellular matrix form the neuro-vascular unit (NVU) [2]. Pericytes cover 60–70% of the abluminal endothelial surface, whereas astrocyte endfeet reach almost 100%, overlaying pericytes [3–5].

Pericytes surround the endothelial walls of capillaries and post-capillary venules and regulate the BBB by promoting the formation of tight junctions and reducing vesicle trafficking in endothelial cells [6]. It was proposed that pericytes can also contribute to the regulation of cerebral blood flow [7], but the mechanisms through which pericytes can achieve this function are still unclear [8, 9]. Strikingly, a recent study showed that acute laser ablation of single pericytes in mice affected capillary dilation but not BBB permeability [10]. The endothelial–pericyte interaction is maintained by the basal lamina and regulated at the cellular level by a machinery of junction proteins arranged in a characteristic “peg-and-socket” myoendothelial junction. The anatomical relationship and close interactions between pericytes and endothelial cells are important for reciprocal regulation of cell adhesion, proliferation, and differentiation via paracrine and juxtacrine signaling through the PDGF-B/PDGFRβ, angiopoetin/Tie2, and TGFβ/TGFβR pathways [6]. Pericyte depletion in mice by genetic mutation of the PDGF-B pathway leads to substantial extravasation of high-molecular substances, including IgG and albumin, due to increased transcellular transport [11, 12]. Interestingly, the increased permeability of the BBB triggered by pericyte loss is heterogeneous throughout the brain, with the cortex and the hippocampus significantly more affected than midbrain regions [13]. These observations suggest that pericyte-independent signals may regulate BBB permeability.

Astrocytes are star-shaped cells attached to penetrating arterial blood vessels and to the outer layer of mature capillaries, where they fulfil supportive functions by maintaining neuronal excitability and homeostasis [14]. Specifically, astrocyte end-feet ensheath the central nervous system (CNS) microvasculature and neuronal synapses and enable the modulation of neuronal activity, BBB formation, and cerebral blood flow. Astrocytes express specialized molecules and release growth factors such as vascular endothelial growth factor (VEGF), glial cell line-derived neurotrophic factor (GDNF), basic fibroblast growth factor (bFGF), and angiopoetin-1 (ANG-1) that are essential for the induction of BBB properties. The specific effects of astrocytes on BBB function have been thoroughly reviewed elsewhere [15–18].

It is now accepted that the BBB is not completely impermeable to large molecules, but that multiple proteins cross the BBB via receptor-mediated transcytosis (RMT). Receptors involved in transcytosis across the BBB include insulin receptor, transferrin receptor, and low-density lipoprotein receptor [19]. Although the extent of protein transcytosis across the BBB is quantitatively lower than in peripheral endothelia, such transport is not negligible, but has instead important physiological roles, as exemplified by insulin [20] and leptin [21]. Importantly, RMT has been exploited by molecular shuttles that enhance the delivery of biologics to the brain [22]. Yet, despite the relevance of RMT for physiological processes and therapeutic development, very little is known about its regulation at the cellular and molecular levels [19, 23, 24]. Here, we highlight the most recent findings on transcytosis across the BBB and its regulation in physiological and pathophysiological conditions.

## Intracellular transport pathways across the blood–brain barrier

**Fig. 1:**
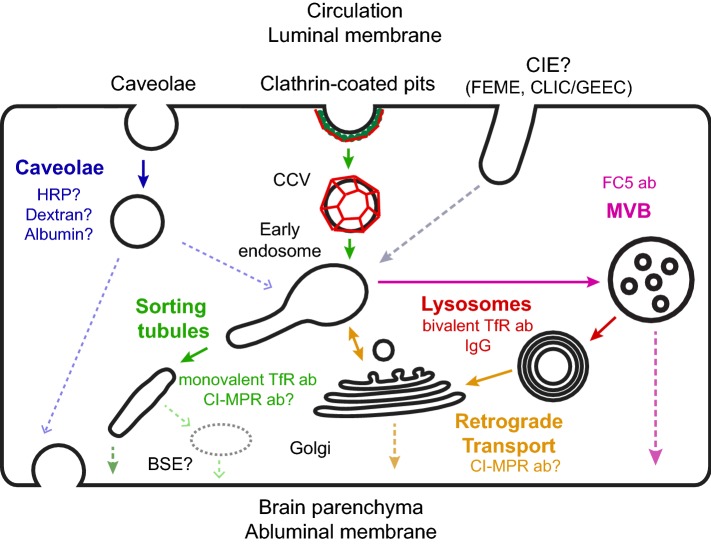
Intracellular transport pathways in brain endothelial cells. Transport across brain endothelial cells can be divided into three distinct processes: internalization, sorting, and exocytosis. For simplicity, only transport from the luminal (apical) to the abluminal (basolateral) membrane is shown, but the same mechanisms may also occur for basolateral-to-apical trafficking. Internalization into brain endothelial cells can occur via caveolae, clathrin-dependent endocytosis, or clathrin-independent endocytosis pathways such as fast endophilin-mediated endocytosis (FEME) or clathrin-independent carriers/glycosylphosphatidylinositol-anchored protein-enriched endocytic compartment (CLIC/GEEC). Internalization pathways converge in the early endosome network, which functions as an intracellular sorting station. Caveolae are thought to undergo transcytosis without fusion with early endosomes to promote transport of receptor-independent molecules (e.g., HRP, dextran, and albumin). Clathrin-coated vesicles (CCV) fuse with early endosomes after shedding the clathrin coat. From early endosomes, cargo can be transported via sorting tubules for transcytosis (e.g., Monovalent TfR and cation-independent mannose-6-phosphate receptor (CI-MPR) antibodies). Alternatively, endosomes mature into late endosomes and multi-vesicular bodies (MVB) which undergo fusion with lysosomes for cargo degradation (e.g., Bivalent TfR antibody, IgG). The retrograde transport pathway can shuttle cargo (e.g., CI-MPR antibody) from lysosomes to the Golgi apparatus and early endosomes. Exocytosis occurs when the sorting/transport compartment fuses with the abluminal membrane and is the least characterized process during endothelial transcytosis. Sorting tubules can fuse directly with the abluminal membrane or they can fuse with an intermediate basolateral sorting endosome (BSE) prior to exocytosis, as observed for transcytosis in epithelial cells. From the Golgi, cargo exocytosis can occur directly by polarized secretion to the abluminal membrane or indirectly by transport to early endosomes and subsequent sorting into tubules. A subpopulation of MVBs is thought to promote transcytosis by fusing with the abluminal membrane and releasing cargo (e.g., FC5 antibody) bound to exosomes. Dotted lines reflect pathways or compartments which have not been experimentally verified

The observations from the landmark electron microscopy study from Reese and Karnovsky [1] led to the paradigm that minimal vesicular transport is a key property of the BBB [25]. While the number of vesicles in BECs is indeed lower than the number of vesicles in peripheral endothelial cells (ECs) [26], by no means is endocytosis negligible at the BBB. On the contrary, electron microscopy studies showed that exogenous horse radish peroxidase (HRP) is internalized, albeit only in low amounts, and transported to lysosomes in BECs [27]. A more recent study also found that endogenous mouse IgG are localized within intracellular vesicles within BECs and transported to lysosomes [28]. Vesicular transport is especially important for RMT across the BBB, as shown for molecular shuttles that enhance antibody delivery to the brain [29]. However, not all internalized antibodies undergo efficient transcytosis. For example, it was shown that both high binding affinity and bivalent antibody binding to the transferrin receptor (TfR) prevented transcytosis and instead, drove transport to the lysosome [30, 31]. The fact that both brain-penetrant (e.g., TfR monovalent antibodies) and non-brain-penetrant molecules (e.g., TfR bivalent antibodies, IgG) are internalized by BECs raise the question to which sorting mechanisms determine successful transcytosis to the brain parenchyma. In this section, we review the known intracellular endocytic mechanisms in endothelial cells and discuss how such mechanisms can regulate transcytosis across the BBB (Fig. 1).

The transcellular transport pathway is initiated by endocytosis at the plasma membrane. For a detailed review on the mechanisms of endocytosis, the reader is referred to recent reviews on the topic [32, 33]. The most highly characterized internalization pathway is clathrin-mediated endocytosis (CME) [34]. A recent study showed that approximately 95% of transmembrane receptors at the plasma membrane are internalized via CME in fibroblasts [35]. Coated pits characteristic of CME can be observed in BECs in brain tissue by electron microscopy [28]. Furthermore, multiple receptors that undergo RMT in BECs require CME for internalization, such as TfR [35, 36] and insulin receptor [37]. Despite the prevalence of CME, other clathrin-independent pathway(s) may also regulate RMT across the BBB.

In peripheral blood vessels, there is strong evidence for the role of caveolae in transcellular transport. Caveolae are 60–80 nm flask-shaped organelles that lack an electron-dense membrane coat and contain oligomeric caveolin-1 [38]. It was previously shown that lung endothelial cells in caveolin-1 deficient mice lack caveolae and exhibit impaired transcytosis of an antibody against aminopeptidase P [39]. More recent data showed that caveolin-1 is also involved in transcytosis across the BBB. Caveolae assembly at the plasma membrane in BECs is negatively regulated by the lipid transporter Mfsd2a [40]. Genetic knock-out of Mfsd2a in mice led to a marked increase in both brain endothelial caveolae and transcellular permeability in the brain vasculature [41]. Abolishing caveolin-1 in Mfsd2a knock-out mice prevented enhanced transcytosis [40]. These results revealed that caveolin-1 is required for the transport of fluid-phase molecules such as dextran and HRP upon Mfsd2a knock-out. Whether caveolin-1 is required for RMT under physiological conditions was not explored. It is important to note that caveolae also regulate lipid transport, mechanoregulation, and cell signaling [42]. It is still unclear whether caveolae are endocytic transport carriers that undergo transcytosis, or instead, they indirectly induce transcytosis via endosomes, for example, through modulation of membrane fluidity. Interestingly, recent studies have challenged the role of caveolae as endocytic transport carriers. First, there is no known specific cargo molecule transported primarily by caveolae [42]. Second, the widespread methods for cholesterol extraction used to disassemble caveolae, for example, treatment with filipin, also affect other clathrin-independent pathways [43, 44]. Finally, knock-out of caveolin-1 upregulated caveolin-independent pathways in mouse fibroblasts [45]. Therefore, further work is required to clarify how caveolae regulate intracellular transport and what is their specific contribution to transcytosis at the BBB.

Further additional clathrin-independent pathways can drive the internalization of receptors, for example, fast endophilin-mediated endocytosis (FEME), macropinocytosis, ultrafast endocytosis, and the clathrin-independent carriers/glycosylphosphatidylinositol-anchored protein-enriched endocytic compartment (CLIC/GEEC) pathway [32, 46]. However, these pathways have been observed only in non-endothelial cell types, and to date, there is no evidence of their presence in BECs.

The internalization pathway can determine the cellular fate of a receptor, as was hypothesized for the LDL receptor which is internalized by both CME and caveolin-dependent endocytosis [47]. However, mechanisms downstream of internalization can also sort receptors to different destinations. One of the best examples of such receptors at the BBB is TfR. Two independent groups showed that monovalent antibodies or bispecific low-affinity constructs against TfR are sorted for transcytosis, while bivalent antibodies or high-affinity constructs against TfR are sorted for degradation at lysosomes [30, 31]. A recent study described that differential sorting of this receptor-antibody pair is regulated by intracellular tubules [48]. Transport via tubules is an efficient and versatile strategy to sort proteins to different intracellular destinations, as it allows separating receptors based on interactions with cytosolic proteins [49, 50] or on the balance between receptor monomers and oligomers [51]. In the case of TfR in BECs, bivalent antibody binding resulted in the formation of macroscopic receptor clusters which failed to enter intracellular tubules [48]. Although tubules were observed both in primary mouse BECs and in mouse brain capillaries in vivo, their molecular identity was not characterized. Work in other cell types showed that endosomal tubules are heterogeneous organelles that can be formed through different membrane-remodeling pathways, including sorting nexins containing the Bin/Amphiphysin/Rvs domain (SNX-BAR), Arf6 GAP proteins with coiled-coil, ankyrin repeats and pleckstrin homology domain (ACAPs) and Eps15 homology domain (EHD) proteins [52]. Therefore, a systematic loss-of-function approach will be required to identify the mechanisms that drive tubule formation and receptor sorting at the BBB.

Sorting by intracellular tubules is likely not exclusive to TfR transcytosis, but could be a general mechanism of transport at the BBB, as suggested by studies on the cation-independent mannose-6-phosphate receptor (CI-MPR). CI-MPR is shuttled by the retrograde transport pathway between the trans-Golgi network and endosomes to deliver lysosomal hydrolases [53]. It was recently found that BECs of porcine and bovine origin express CI-MPR and that this receptor is able to undergo bidirectional apico-basal trafficking across polarized BECs [54]. Interestingly, recent work in HeLa cells showed that CI-MPR transport from endosomes occurs via intracellular tubules formed by the SNX1/2-5/6 complexes [55]. Whether SNX-dependent tubules are responsible for the sorting and apico-basal trafficking of CI-MPR and transcytosis of TfR at the BBB still needs to be investigated.

Additional evidence supporting the relevance of intracellular sorting for transcytosis across the BBB comes from the extensive characterization of the novel camelid antibody construct FC5. FC5 crosses the BBB via RMT driven by the putative receptor Tmem30 [56, 57]. By performing multi-plexed mass spectrometry on purified organelle fractions from an immortalized rat BEC cell line, it was shown that FC5 was enriched in early endosomes and a subpopulation of multi-vesicular bodies (MVBs). On the other hand, a construct that does not undergo transcytosis in vivo was enriched in late endosomes/lysosomes and depleted from early endosomes [58]. These observations strengthen the hypothesis that the lysosomal network is an additional mechanism to restrict transcellular transport of blood-borne proteins [27, 28]. The authors of this study [58] proposed a new pathway for FC5 transcytosis which could be mediated by MVB fusion with the plasma membrane, as known to occur during exosome biogenesis [59].

The last step during transcellular transport at the BBB is exocytosis, which refers to the fusion of the carrier organelle with the opposite membrane. It is still unknown whether tubules and/or MVBs directly undergo fusion with the abluminal or luminal membrane. An alternative scenario is that tubules and/or MVBs may act as transport intermediates between apical and basolateral endosomes, as observed in epithelial cells [60]. Recently, an assay based on total internal reflection fluorescence (TIRF) microscopy was developed to better characterize insulin and LDL transcytosis in endothelial cells [61]. The application of this or other similar novel quantitative approaches to BECs will be instrumental to shed light on the mechanisms regulating exocytosis at the BBB. Although most studies have focused on apical-to-basolateral BBB transport (i.e., influx to the brain parenchyma), trafficking from the basolateral to apical membrane (i.e., efflux from the brain parenchyma) is very likely occurring within BECs. Recent work suggested that basolateral-to-apical transport of LRP1 receptor may play an important role for efflux and clearance of amyloid β (Aβ) [62]. However, data on this pathway on BECs are scarce and it is still unclear whether the same mechanisms regulating apical-to-basolateral transport also operate in the opposite direction.

Transcellular transport across the BBB is currently being exploited to enhance delivery of therapeutic antibodies to the brain. Utilizing the TfR pathway as a shuttle mechanism has proved to be a successful strategy for brain delivery in preclinical species [30, 63]. Key lessons from these studies were that the extent of transport to the brain parenchyma is regulated by TfR-antibody avidity and affinity. These results were recently confirmed using TfR-targeted nanoparticles [64]. Interestingly, the effects of avidity and affinity on transport were not observed when different antibody formats against TfR [65] or antibodies against another receptor [57] were used as shuttles. These findings underscore the fact that transport across the BBB is not solely dependent on antibody binding properties, but is likely receptor- and epitope-specific. Recently, a proteomic analysis of mouse brain endothelial cells was performed to identify novel BBB-specific membrane proteins and increase the repertoire of transcytosis shuttles. This work identified that the transmembrane proteins CD98hc and Glut1 are highly enriched in brain capillaries [66]. Antibodies against CD98hc substantially increased transport across the BBB in mice after systemic dosing. However, it should be noted that although CD98hc- and TfR-based shuttles can enhance transport of biologics to the brain in preclinical species, the safety, efficacy, and pharmacokinetic profiles of these molecules are likely impacted by the ubiquitous expression of TfR and CD98hc in peripheral tissues. An alternative approach to discover brain-specific shuttles is to screen antibody or peptide libraries for molecules that can cross the BBB in preclinical species. With this method, two different groups identified new binders with enhanced brain penetration after systemic dosing [67, 68]. While promising, these novel shuttles rely on poorly characterized receptors (e.g., CD98hc, Tmem30) with unknown intracellular transport routes. As discussed below, we consider that optimization of new shuttles will require a thorough characterization of their intracellular transport pathways. While more work is required for a better understanding of how receptors can cross the BBB, the studies summarized in this section highlight the relevance of intracellular trafficking for transcytosis as well as its potential to be exploited as a delivery mechanism of therapeutic antibodies. In the next section, we discuss how intracellular transport at the BBB is affected during disease.

## Regulation of intracellular transport across the BBB during disease

Sterile brain injuries (e.g., status epilepticus, non-penetrating trauma, and vascular accident) and chronic stages of diverse brain diseases are often associated with capillary damage and increased BBB permeability [69, 70]. The inflammatory response that accompanies the progression of numerous CNS disorders promotes an aberrant neuro-vascular remodeling resulting in neuronal dysfunction and neurodegeneration [71–73]. There is ample evidence to demonstrate that inflammation opens tight junctions and enhances paracellular transport, which has been reviewed elsewhere [2]. Here, we focus on the specific changes to intracellular transport across the BBB. In particular, numerous electron microscopy (EM) studies have found an increase in the density of intracellular vesicles in BECs after various forms of injury to the BBB [reviewed in 74]. Such vesicles are partly elongated, form vesiculo-tubular structures, and reflect changes in the intracellular endocytic network within BECs. A high number of vesicles and vesiculo-tubular structures is often assumed to reflect an increase in transcytosis rate. However, this conclusion is based on correlative evidence and requires functional validation, as it offers no insight into the transport of cargo molecules. To overcome this limitation, an alternative method was recently developed to investigate the intracellular distribution of endogenous and exogenous cargo molecules at the BBB [28]. The authors used high-resolution ex vivo confocal microscopy to detect intracellular accumulation of endogenous IgG within vesicles in mouse BECs. Strikingly, increased BBB permeability led to a significant reduction in the number of IgG-positive vesicles, likely due to increased fusion with the abluminal membrane and accelerated IgG delivery to the brain parenchyma. This sensitive method will facilitate the functional characterization of intracellular transport at the BBB during brain injury or disease. Below, we highlight recent examples of intracellular transport mis-regulation and its potential consequences in CNS disorders.

In the context of Alzheimer’s disease (AD), transcytosis across the BBB is increasingly investigated, as it is one of the mechanisms responsible for both clearance of Aβ from the brain and for delivery of therapeutic antibodies to the brain. Genome-wide association studies have linked the disease to several genes that may be involved in Aβ clearance [75]. Interestingly, at least two of them, PICALM and CD2AP, are expressed in BECs and are involved in endocytosis [62, 76]. The function of PICALM in Aβ transport has been studied in considerable detail. PICALM is a ubiquitously expressed clathrin adaptor protein which closely interacts with LRP1, a key player in receptor-mediated endocytosis and Aβ efflux from the brain [77, 78]. After binding to LRP1 in an Aβ-dependent manner, PICALM facilitates trafficking to Rab5- and EEA1-positive early endosomes and to Rab11-positive recycling endosomes [62]. A single-nucleotide polymorphism in the *Picalm* locus that leads to a lower *Picalm* expression is associated with AD, in agreement with the concept that PICALM is involved in the transendothelial transport and efflux of Aβ [62].

In addition to disturbed PICALM-mediated transcytotic efflux of Aβ, there is also evidence that transcytotic influx of plasma proteins into the brain is enhanced in AD and may contribute to disease pathology [reviewed in 79]. It was proposed that Aβ can induce apoptotic cell death of pericytes in the hippocampus leading to vascular regression, reduction of nutrient transport and energy substrates to neurons, as well as extravasation of blood components in the brain parenchyma. The combination of these multiple factors thus contributes to neuroinflammation and neuronal injury, altogether promoting neurodegeneration [73, 80, 81]. However, the extent of transcytosis during AD seems to be markedly lower than in neuroinflammatory diseases and the specific changes to intracellular transport pathways are still unknown. Interestingly, in mouse models of AD, endogenous plasma proteins as well as exogenously administered therapeutic antibodies were transported to the brain parenchyma, but there was no evidence for a widespread disruption of the BBB [79, 82, 83]. This result suggests that the BBB is a hurdle for therapeutic antibodies in AD unless other features increase passage.

In stroke patients, EM also identified an increase of vesiculo-tubular structures within BECs [84]. Cerebral ischemia leads to an upregulation of caveolin-1 and caveolin-2 in BECs but not in other cell types of the brain [85]. As early as 6 h after ischemia, there was an increase in endothelial caveolae and albumin uptake/transport to the brain parenchyma [86]. In accordance with the key role of caveolin-1 in caveolae formation, caveolin-1 deficient mice exhibited reduced albumin uptake/transport to the brain parenchyma in the first hours after stroke. At later timepoints, the BBB was disrupted due to disturbed tight junctions, but this process did not depend on caveolin-1 [86]. The relevance of altered intracellular transport for the post-ischemic BBB disruption is supported by data showing a parallel increase of uncoated vesicles and BBB disruption in obese mice [87]. Hypertension but not old age further increased the number of post-ischemic endothelial vesicles [88, 89]. The mechanisms by which obesity or hypertension promote post-ischemic transcytosis are still unclear. In addition to the general increase in intracellular transport, cerebral ischemia may exert very specific effects on the machinery involved in RMT, as illustrated by the upregulation of TfR [90], which may enable the efficient delivery of therapeutic antibodies via this pathway.

Altered intracellular transport was also observed for multiple sclerosis (MS) patients. The number of intracellular vesicles within BECs correlated with disease severity [91]. Interestingly, while there was a substantial increase in non-coated intracellular vesicles between patients, no differences in the number of coated vesicles was detected, suggesting that different endocytic pathways are independently regulated. The findings on increased intracellular vesicles are reproduced in the experimental autoimmune encephalomyelitis (EAE) animal model of MS [92]. Caveolin-1 and caveolin-2 levels are elevated in endothelial cells during EAE [93, 94], possibly because the pro-inflammatory transcription factor NF-κB controls expression of caveolin-1 [95]. In addition, inflammatory cytokines likely present in MS lesions are also known to stimulate fluid-phase endocytosis [96–98]. In contrast, Wnt signaling inhibits caveolin-1 expression, an effect that may contribute to the central role of Wnt in the development and maintenance of the BBB [99]. Similar to its effect in stroke models, deficiency of caveolin-1 significantly mitigated the course of EAE [93, 100] and reduced the albumin uptake by endothelial cells [100]. However, the latter effect was lost if mice were matched for the EAE severity score. As discussed in the previous section, this observation could be explained by the upregulation of caveolin-independent endocytosis upon caveolae disassembly. Interestingly, caveolin-1 deficiency reduced clinical severity in the EAE model of MS by preventing transcellular migration of Th1 lymphocytes to the CNS [93, 100]. Caveolae are rich in VCAM-1 that binds to integrins on the surface of Th1 cells, suggesting the intriguing possibility that Th1 cells use the endosomal network as a route for their transcellular migration across the BBB.

The changes in BBB permeability observed during injury or disease are mediated by different cell types. Microglial cells, the resident macrophages in the CNS, are the primary cellular regulators of the inflammatory response in the brain [101, 102]. Upon brain injury, microglia synthesize a plethora of pro-inflammatory cytokines which can directly affect BBB permeability [103]. A more general description of the key roles of microglia during injury and disease and their general effect on BBB permeability is out of the scope of this review, but has been comprehensively reviewed elsewhere [101–103]. Altered BBB permeability during disease can also be mediated by pericytes and astrocytes, as these cells display immune properties and participate in both innate and adaptative immunity processes in vivo [104]. Changes of the pericyte inflammatory profiles have been reported in CNS pathologies associated with ischemic stroke [105], AD [81, 106], and traumatic brain injury [107]. For example, pericytes can release matrix metalloproteinases (e.g., MMP9) upon TNF-α stimulation impacting the integrity of the BBB. Pericytes constitutively express major histocompatibility complex (MHC) class I but not MHC class II or the co-stimulatory molecules CD80 or CD86. In vivo, they can secrete chemokines and cytokines and attract immune cells to the site of inflammation in the presence of inflammatory cytokines including IFN-γ, IL-17, TNF-α, and IL-1β [104]. In the case of astrocyte-mediated regulation, the arachidonic acid (AA) pathway emerged recently as a regulator of neuro-vascular coupling at the capillary level [108]. This pathway depends on astrocytic Ca^2+^ signaling and the production of AA-derived prostaglandins (PGs) [109]. The AA pathway has also been involved in the regulation of pericyte tone and diameter, which is indicative of its importance for the regulation of the neuro-vascular unit [6]. In astrocytes, production of the pro-inflammatory PGs depends on the activity of the diacylglycerol lipase (DAGL) and the monoacylglycerol lipase (MAGL) [108, 110, 111]. In the brain, MAGL is the main enzyme responsible for the production of AA and PGs through the cleavage of the main endocannabinoid 2-AG. Specific deletion of MAGL expression in astrocytes significantly increases 2-AG levels while concomitantly reducing AA levels and protecting against neuroinflammation [110, 111]. Inflammation drives the activation of the neurovasculature, thus disrupting the integrity of the BBB and substantially inducing its permeability as reported in several neurodegenerative diseases. Inhibition of MAGL activity, and, therefore, modulation of PGs expression were recently shown to prevent an inflammation-driven increase in BBB permeability [112] suggesting a dual role of the AA pathway controlling neuro-vascular coupling and BBB integrity/permeability. However, it should be stressed that changes to the BBB upon immune challenges are not restricted to intracellular transport but also affect paracellular transport and immune cell transmigration. Therefore, additional work is required to identify the signaling pathways that specifically mediate changes to the endosomal network during disease. Nonetheless, the examples summarized here provide some evidence that alterations to intracellular transport at the BBB may play a key role in the pathophysiology of CNS disorders.

## Open questions and challenges

The recent findings on intracellular transport across the BBB raise new important questions. How many pathways can drive transcytosis (e.g., tubules from early endosomes, MVBs, retrograde transport, caveolae)? What is the relative contribution of each pathway to overall transport into and out of the brain? What mechanisms determine which pathway(s) will sort different receptors? How do these processes contribute or respond to disease? Answering these questions first requires identifying the molecular mechanisms controlling intracellular transport at the BBB. Such mechanisms may be unique to endothelial cells, as BECs do not express known regulators of epithelial transcytosis, such as Rab17 and Rab25 [48]. We consider that to improve the current understanding of BBB transcytosis and reveal the specific mechanisms of intracellular transport within BECs, it is necessary to adopt emerging technologies from the field of molecular cell biology, such as super-resolution microscopy, unbiased image analysis, and genome editing. The value of quantitative microscopy/image analysis for the BBB is highlighted by the findings on new mechanisms of transferrin receptor sorting [48] and on the regulation of the endo-lysosomal network of porcine BECs by astrocytes [113]. However, a major limitation of these studies is that they relied on two-dimensional cellular models that do not fully recapitulate the complex cellular architecture of the NVU. Moreover, most studies on the BBB use animal models which may not be translatable to human physiology. A solution to these caveats may come from the use of increasingly complex in vitro human BBB models, including multicellular human BBB spheroids [114], inducible pluripotent stem-cell-derived multicellular co-cultures [115, 116], and microphysiological systems with fluid flow [117]. The study of such complex models requires the adoption of new imaging technologies [118] that allow the acquisition of the complete NVU volume with subcellular resolution at endocytosis-relevant time scales. The combination of improved multicellular BBB models with advanced microscopy imaging will likely reveal physiologically relevant mechanisms for the regulation of intracellular transport across the BBB.

We propose that a better understanding of intracellular transport within BECs will lead to improved therapies for CNS disorders, specifically through the impact in three key areas of drug development. First, a thorough characterization of transcytosis across the BBB and its alterations during disease can point to new therapeutic targets. For example, upregulation of wild-type PICALM in BECs may help decrease Aβ levels in AD. Second, detailed knowledge on the regulation of intracellular transport will contribute to rational design of antibody shuttles. Current shuttle engineering focuses mostly on modifying binding properties, but as discussed above, target binding does not always correlate with transport across the BBB. By mapping which intracellular routes are most efficient for transcytosis, shuttles with desired trafficking profiles could be engineered, for example, by modifying net protein charge [119] or by adding glycosphingolipids that regulate sorting [120]. Third, understanding the itinerary of proteins within BECs will improve target receptor selection and molecule optimization. Selection criteria are now primarily based on receptor expression and specificity. However, abundant receptors in BECs are not necessarily the most efficient for transcytosis, as is the case for TfR [121]. During molecule optimization, another critical factor to consider is the interaction between a given shuttle and other endosome-resident proteins in the same pathway. For example, it is not yet known whether FcRn, an endosome-resident receptor expressed in BECs which specializes in recycling antibodies back to the circulation [122], impacts transcytosis of TfR-antibody-based shuttles. Furthermore, the development of new antibody formats using, for example, pH-sensitive linkers [123] or sequential targeting to promote binding at the cell surface followed by intracellular redirection [29], clearly require a detailed understanding of sorting pathways and protein interactions within endosomes. Through the impact on these three areas, we consider that investigating the mechanisms of intracellular pathways at the BBB will help to accelerate the clinical development of new brain delivery platforms for biologics.

The evidence on intracellular transport across the BBB presented in this review demonstrate that, instead of being cells with “reduced vesicular transport”, BECs possess a complex intracellular transport network controlling receptor transport to the brain. Characterizing these pathways will help drive a renaissance in the field of intracellular transport across the BBB.
